# Supplementary material for: Cancer patient survival can be parametrized to improve trial precision and reveal time-dependent therapeutic effects
Source: Nat Commun. 2022 Feb 15;13:873. doi: 10.1038/s41467-022-28410-9 (PMC8847344; doi:10.1038/s41467-022-28410-9)
Supplement: Supplementary file 5 — Supplementary Dataset 2 [file 41467_2022_28410_MOESM5_ESM.zip › Supplementary Data File 2/Weibull all fits survival plots- Fig 1, 2, S1, S2/ATTENTION_2B_placebo.pdf]

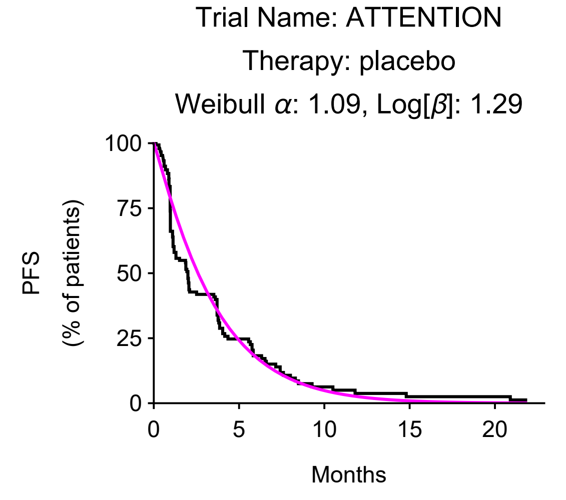
Trial Name: ATTENTION
Therapy: placebo
Weibull $\alpha$: 1.09, Log[$\beta$]: 1.29
PFS
(% of patients)
100
75
50
25
0
0
5
10
15
20
Months
